# Helicopter Air Transport of a Patient Using High Flow Nasal Cannula: A Case Report

**DOI:** 10.7759/cureus.41317

**Published:** 2023-07-03

**Authors:** Francisco Das Neves Coelho, Rui Alves, Carlos Raposo

**Affiliations:** 1 Helicopter Emergency Medical Services, Instituto Nacional de Emergencia Medica, Lisboa, PRT; 2 Intensive Care, Hospital Egas Moniz, Centro Hospitalar Lisboa Ocidental, Lisboa, PRT

**Keywords:** air transport, aeromedical transport, hems, interhospital transport, patient rescue, out-of-hospital, operative strategy, non-invasive ventilaton, helicopter ems

## Abstract

The classic out-of-hospital approach to a patient with severe acute respiratory failure involves both orotracheal intubation and invasive mechanical ventilation. The use of non-invasive methods for respiratory support has been shown to be beneficial in managing both acute and chronic respiratory failure. However, its use had not been previously considered for air medical transport due to concerns related to airway safety during flight, limited oxygen availability, and limited experience in this setting.

We describe the successful inter-hospital helicopter transport of a patient with end-stage lung disease to a transplantation unit while utilizing a high-flow oxygen cannula, which was performed without significant complications. Our successful case report raises the possibility that high-flow nasal cannulas may be safely employed in the management of respiratory failure in specific patient populations during air medical transport.

## Introduction

Air-medical transportation of critically ill patients poses specific challenges due to the inherent depressurization that occurs at high altitudes. As the aircraft ascends without pressurization, the partial pressure of oxygen (pO2) decreases compared to sea level, leading to a reduced effective FiO2 despite a constant oxygen concentration in the atmosphere. This phenomenon is known as hypobaric hypoxia [1]. Studies have shown a sustained decrease in inspired pO2 proportional to the flight altitude, with greater clinical significance observed in non-pressurized flights above 6000 feet [2]. However, critically ill patients requiring helicopter evacuation often have conditions that reduce their physiological reserve, making them less tolerant to acute altitude exposure [3].

Managing oxygen capacity is also a concern for medical teams during the transport of patients with respiratory failure. The calculation for the duration of oxygen reserves for a specific transport can be performed using the following formula:

$O_{2} Reserves (min)= \frac{Capacity (L) \times Pressure (Bar)}{O_{2} Flow(L/min))}$

This formula allows for accurate quantification of the required oxygen amount for a specific transport when patients receive a fixed oxygen flow through a flowmeter. For critically ill patients, oxygen delivery is often based on a fixed percentage of airflow. Although the airflow delivered to the patient may vary, the estimated oxygen consumption can be calculated by modifying the formula as follows:

$O_{2} Reserves (min)= \frac{Capacity (L) \times Pressure (Bar)}{Minute Volume(L/min)\times FiO_{2}}$

When preparing for transport, it becomes necessary to calculate the duration of oxygen reserves for the planned journey. Additionally, it is recommended to add a supplemental oxygen reserve to account for any unexpected incidents during the transport, such as increased oxygen demand from the patient, unexpected delays, or leaks in the oxygen delivery system [4].

The use of high-flow oxygen cannulas (HFOC) has seen progressive growth in dedicated medical wards for respiratory patient care, with a positive impact on managing acute respiratory failure (ARF). Recently, HFOC has also been employed in domiciliary care for patients with chronic obstructive pulmonary disease (COPD) requiring long-term respiratory support, resulting in improved therapeutic adherence and quality of life [5-7].

The use of non-invasive methods of respiratory support in the pre-hospital context, particularly in helicopter transport, has not been extensively explored. Concerns regarding potential exacerbation of acute respiratory failure during flight in an unpressurized cabin, limited capacity for safe in-flight endotracheal intubation, limited oxygen supplies, and logistical challenges associated with using the device during transport have limited its use. However, the increasing use of non-invasive respiratory support in both the hospital and home settings will expose medical emergency teams to patients using such devices. In specific situations, maintaining patients under the same respiratory support during transport may provide benefits.

## Case presentation

The patient was a 49-year-old male. He had a relevant history of alveolar silico-proteinosis for 13 years, and required multiple hospital admissions and interventions due to progressive worsening of his underlying condition. The patient had already undergone two total lung lavages in the past under invasive mechanical ventilation and extracorporeal oxygenation support. He also received supra-selective embolization of pulmonary vessels for uncontrolled hemoptysis. Additionally, he has had multiple recent hospitalizations for exacerbation of his respiratory disease over the past 18 months. While at home, the patient depended on HFOC with a flow rate of 45L/min and FiO2 of 42% for adequate oxygenation. He was actively listed for lung transplantation and had no history of smoking, alcohol, or substance abuse. There were no other significant clinical or therapeutic backgrounds.

The patient was admitted to the pulmonology department of a local hospital due to progressively worsening dyspnea over the three weeks before admission, accompanied by a complete loss of exercise tolerance. An extensive study was conducted during his hospital stay, which showed stability in respiratory pathological findings and no radiologic evidence of infectious lung disease. Transthoracic echocardiogram results did not suggest acute pulmonary hypertension. Clinically, the patient showed slight symptomatic improvement with methylprednisolone 1mg/kg/day, but marked exercise intolerance persisted. Arterial blood gas analysis revealed reasonable oxygenation with his usual HFOC, which remained stable throughout the last week of hospitalization.

The final diagnosis made by the attending team was an exacerbation of symptoms due to the progression of chronic lung disease. Consequently, the patient was accepted by the lung transplantation department. It is important to note that the same center declined ventilatory support or extracorporeal oxygenation as a bridge to transplantation, which was a prerequisite for patient admission. Considering the long distance between the hospitals by road and the patient's heavy dependence on HFOC, the safety of land transportation was questioned due to its longer duration, limitations in the oxygen autonomy of ground vehicles, and patient comfort. Given that air transport would be the fastest method for this patient, the medical team contacted the National Institute of Medical Emergency (NIME) to inquire about the possibility of performing the transport by air. Our AgustaWestland 139 helicopter was chosen for this mission due to its flight autonomy and oxygen supplies. An estimated transport time of 75 minutes was calculated between the landing pads of both hospitals.

Our emergency medical helicopter has a conventional oxygen capacity consisting of three 5L cylinders and one 3L cylinder, with a maximum capacity of 200 atmospheres (total capacity: 3600L). Based on the calculation formulas for oxygen autonomy, the baseline oxygen requirement at the departing hospital was calculated to be 21L/min. For this volume, a fully loaded 5L cylinder would provide 48 minutes of oxygen. Considering the possibility of respiratory deterioration during transport, we accounted for up to 20% of the patient's baseline oxygen consumption at the originating location. Therefore, we estimated an oxygen consumption based on a hypothetical flow rate of 60L/min and an FiO2 of 60%, resulting in an oxygen usage of 36L/min. For this volume, a fully loaded 5L cylinder would provide 28 minutes of oxygen. Additionally, we included an additional 50% oxygen reserve to ensure the patient's needs in case of unexpected events during the journey and to allow for airway management in case of critical complications during transport. In total, we transported a total of eight 5L cylinders at maximum capacity (total capacity: 8000L).

High-flow therapy is not part of the therapeutic options available in our NIME helicopters. To ensure the patient's oxygenation, the pulmonology service temporarily provided the use of their Philips® Respironics V60 (Philips, Amsterdam, the Netherlands) equipment and Fisher & Paykel® MR850 (Fisher & Paykel Healthcare, Auckland, New Zealand) humidifier for this transport. As contingency plans in case of severe hypoxia during aeromedical transport, we planned to switch from high-flow therapy to positive pressure ventilation (CPAP or B-level), or if necessary, convert to invasive mechanical ventilation using a laryngeal mask. Prior to transport, we contacted the receiving unit in advance and alerted them to the possibility of ICU admission in the event of respiratory decompensation during transport.

**Figure 1:**
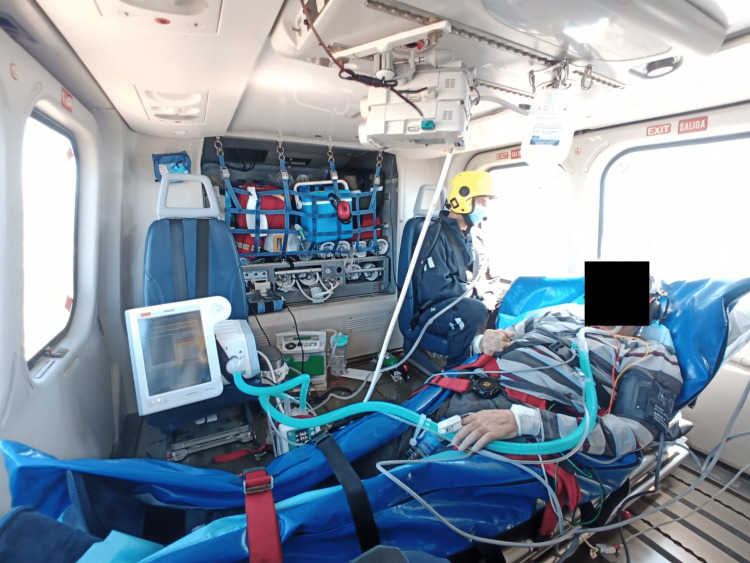
AgustaWestland (AW) 139 medicalised helicopter cell during patient transportation to transplantation unit.

The total duration of the mission was 141 minutes, with 82 minutes spent in flight. The helicopter transport route was conducted at an altitude of 1000 to 2000 feet, which is considered low altitude flight. Vital signs were monitored before, during, and after transport, and the corresponding blood gas analyses are summarized in Table 1 and Table 2. The helicopter cabin setup is demonstrated in Figure 1. The transport proceeded without clinical complications. The FiO2 needed to be increased to 50% during the flight to maintain the patient's baseline oxygen saturation levels, but the flow did not need to be increased during transport. Two incidents related to the air humidifier occurred during the transport. The first incident was the detection of a malfunction in the humidification chamber during the equipment check before departure, which was replaced with a WILAmed® AIRcon Gen2 humidifier. The second incident was the loss of function of the humidification chamber due to flooding of the system during takeoff and altitude gain, caused by the loss of pressurization in the aircraft's cabin, rendering it unusable. However, these incidents did not compromise the safety of the transport. In total, two 5L oxygen cylinders from the aircraft were used (2000L of oxygen).

**Table 1:** Vital signs of the patient under High-Flow Nasal Cannula at various moments during helicopter transport RR – respiratory rate, CPM – cycles per minute, SpO2 – peripheral O2 saturation, HR – heart rate, bpm – beats per minute, NIBP – non-invasive blood pressure, mmHg – millimeters of mercury

Time	08:33	10:19	10:49	11:14	11:41	12:05
Place	Pulmonology (departure)	Helicopter (take-off)	Helicopter (in-flight)	Helicopter (in-flight)	Helicopter (after landing)	Transplantation unit
Flow	45L/min	45L/min	45L/min	45L/min	45L/min	45L/min
FiO2	45%	45%	50%	50%	50%	50%
RR (cpm)	22	27	24	20	24	19
SpO2 (%)	93%	91%	95%	94%	95%	96%
HR (bpm)	78	95	89	72	79	98
Rhythm	Sinus	Sinus	Sinus	Sinus	Sinus	Sinus
NIBP (mmHg)	145/89	153/102	149/99	143/99	144/95	151/101

**Table 2:** Arterial blood gas samples of the patient before and after flight BE: Base Excess

Time	07:59	12:15
Place	Pulmonology (before departure)	Transplantation Unit (after admission)
FiO2	45%	50%
pH	7.45	7.43
pCO2 (mmHg)	46.7	48.3
pO2 (mmHg)	78.9	146
HCO3- (mmol/L)	33.1	32.2
SO2 (%)	94.9%	100%
BE (mmol/L)	7.4	6.6
Lactate (mmol/L)	1.4	1.9

## Discussion

The increasing diversification of oxygen administration systems defies the classical approach for patients with ARF during air medical transport. Flight physiology and safety manuals recommend cardiopulmonary stabilization before takeoff. For patients with ARF, this stabilization often involves orotracheal intubation and initiation of mechanical ventilatory support while the patient is still on the ground. This is because the logistics of airway stabilization and ventilation optimization during helicopter transport are virtually impossible due to the limited space inside the aircraft cabin, which hinders a safe approach to these patients during flight. The excess noise also limits communication between staff and tracheal tube confirmation by auscultation.

In a hospital setting, the limited availability of staff proficient in providing appropriate mechanical ventilation, as well as the potential consequences associated with such support for critically ill patients, has driven the development of alternatives to invasive mechanical ventilation. Currently, non-invasive ventilation (NIV) and HFOC provide advanced solutions widely used in patients with acute or chronic respiratory failure. In patients with ARF or exacerbated chronic respiratory failure, the use of these methods for respiratory support is associated with a reduced need for mechanical ventilation. Moreover, their simplified interface and reduced learning curve enable their use in any hospital area dedicated to the treatment of acute patients [5,8].

The feasibility and safety issues regarding the use of such devices have been investigated by several groups. The most comprehensive work exploring the use of NIV during air transport comes from an Australian series, which describes its use in air transport in 128 non-trauma patients, of whom 65% were already receiving this ventilatory support. The average duration of air transport was 65 minutes, during which only two patients were unable to tolerate the use of NIV, one due to worsened consciousness and the other due to mask intolerance [9]. Two working groups explored the use of NIV as the primary approach to non-trauma patients. An Australian group studied its use in the primary approach and air transport of 106 non-trauma patients, where respiratory failure was successfully managed with NIV in 81% of patients. Failure to manage respiratory failure with NIV was identified in 19% of patients, and failure to adapt to NIV was associated with a longer delay to the hospital [10]. A Spanish group compared the use of NIV in the stabilization and transport of patients evacuated by helicopter due to acute respiratory failure versus standard medical treatment in 40 patients, and the use of NIV during the flight did not present complications and was associated with a lower risk of orotracheal intubation and lower oxygen consumption during transport [11]. An American group described the successful use of continuous positive air pressure using a helmet-type device in ten patients [12]. Although helmet-type devices can be adapted to high-flow oxygen flow regulators (similar to those used by high-flow devices), this working group used helmet devices paired with a Hamilton® T1 (Hamilton Medical, Bonaduz, Switzerland) transport ventilator.

In our review of the literature, we found only one helicopter transport performed under similar conditions, as described by a US air medical transport group. They reported the transport of a 49-year-old man with SARS-CoV-2 pneumonia on HFOC from a smaller hospital to an ECMO center. The reason for non-ventilation in that case was due to the patient's expressed desire not to receive mechanical ventilatory support [13].

Despite limited experience in their use, non-invasive methods of oxygenation appear to be safe and effective for air transport of non-traumatic critically ill patients. However, it is important to consider the particularities of air medical transport when using such devices. HFOC systems were not tested in unpressurized conditions, and it is unknown whether oxygen delivery in-flight is as reliable as in ground conditions. HFOC humidifiers are not designed to withstand significant pressure differences in their environment, and depressurization may lead to system flooding and subsequent loss of function. This can be prevented by disconnecting the circuit so that in-system and atmospheric pressures may equalize. It's important to assess whether the aircraft used allows for an adequate airway approach since ARF may worsen during flight, and the anticipated backup plan would involve orotracheal intubation. Oxygen economics play an important role, since oxygen consumption is increased with non-invasive methods of oxygenation. It is also important to consider whether rescue teams are properly equipped and trained in the use of NIV or HFOC, as well as the proper accommodation and maintenance of specific equipment. Limited experience of emergency teams in the use of such equipment restricts their use in the field. It’s also important to remember that, given the pathophysiological differences in ARF between medical and trauma patients, neither NIV nor HFOC are recommended as the first approach for a trauma patient.

Lastly, in patients with infectious respiratory diseases at high risk of contagion, orotracheal intubation and the use of a ventilatory circuit with enhanced filters may still be indicated to minimize the risk of contagion. This topic has gained relevance during the SARS-CoV-2 pandemic, where the need to transport patients with viral pneumonia has presented a greater number of technical and logistical challenges to ensure their safe air transport [14].

The responsibility of considering the risks and benefits associated with helicopter transport of patients using non-invasive methods of respiratory support lies with the attending physician. In our report, we deemed the use of HFOC during the helicopter transport to be safe, considering that the patient's respiratory condition was chronic and had been stable for several days prior to the transport. The patient's oxygen requirements at the referring hospital were identical to those used at home. The planning of the helicopter transport among the entire team (physician, nurse, and pilots) allowed the team to share the same mental model, which greatly contributed to the anticipation of complications during the flight.

## Conclusions

We described the planning, execution, and complications of the helicopter transport of a patient with severe chronic lung disease undergoing high-flow oxygen therapy in preparation for lung transplantation. To our knowledge, this was the second successful helicopter transport performed and reported under such circumstances.

Air medical transport of patients under this type of respiratory support is feasible and appears to be safe. Similar to other complex transports of critically ill patients, the patient's route should be adequately planned, and proper logistics related to transport in an air vehicle with an unpressurized chamber should be considered. It is the responsibility of the medical emergency team to judiciously assess the risks and benefits associated with transporting patients using non-invasive methods of respiratory support based on their experience and the best interests of the patient.
